# Temporal Machine Learning Models for Classifying Suspected Dengue Cases in Mexico Using Surveillance Data from 2025

**DOI:** 10.3390/diseases14050155

**Published:** 2026-04-28

**Authors:** Jorge Soria-Cruz, Enrique Luna-Ramírez, Iván Castillo-Zúñiga, Jaime Iván López-Veyna, Ma. Angélica Estrada-Ramírez, Juan Antonio González-Morales

**Affiliations:** 1 Campus El Llano Aguascalientes, Tecnológico Nacional de Mexico, Km. 18 Carretera Ags.-S.L.P., Aguascalientes 20330, Mexico; jorge.sc@llano.tecnm.mx (J.S.-C.); ivan.cz@llano.tecnm.mx (I.C.-Z.); angelica.er@llano.tecnm.mx (M.A.E.-R.); juan.gm@llano.tecnm.mx (J.A.G.-M.); 2 Campus Zacatecas, Tecnológico Nacional de Mexico, Carr. Panamericana Entronque a Guadalajara s/n, Col. La Escondida, Zacatecas 98000, Mexico; ivanlopezveyna@zacatecas.tecnm.mx

**Keywords:** Mexico, dengue, machine learning, binary classification, temporal validation, threshold optimization, performance metrics, predictive modeling, epidemiological surveillance

## Abstract

**Background**: Dengue fever remains a major public health challenge in Mexico, exhibiting pronounced seasonal behavior and substantial geographic heterogeneity. Using recent epidemiological surveillance data may improve predictive performance and better reflect the current epidemiological context. **Objective**: The aim of this study was to develop and compare temporal machine learning models for the binary classification of confirmed and negative dengue cases in Mexico using 2025 national surveillance data. **Methods**: A total of 68,222 suspected dengue cases reported in 2025 were analyzed. The outcome variable was CASE_STATUS, encoded as 0 for negative cases and 1 for confirmed cases. The dataset was divided chronologically into training (January–September), validation (October), and testing (November–December) subsets. Nine machine learning algorithms were evaluated: Random Forest, Bayesian Network, XGBoost, CatBoost, Naïve Bayes, Logistic Regression, Multilayer Perceptron, Support Vector Machine, and LightGBM. Preprocessing included scaling, encoding, age discretization for Bayesian Network, class imbalance treatment, and model-specific feature-importance analyses. Performance was assessed using accuracy, Precision, Recall, F1-score, ROC-AUC, and PR-AUC. **Results**: Random Forest achieved the best overall performance, with the highest test F1-score (0.7254) and PR-AUC (0.7300) at an optimized threshold of 0.397, together with a high Recall (0.8938). Bayesian Network achieved the highest test accuracy (0.7023) and ROC-AUC (0.7756), although its overall operational balance was less favorable considering class imbalance. Geographic and institutional variables were the most influential predictors across models, whereas comorbidities generally contributed less. **Conclusions**: Temporal machine learning models are useful for dengue case classification in Mexico, and Random Forest was the most robust approach, balancing sensitivity and overall predictive performance. From an operational perspective, this finding is especially relevant in dengue surveillance, where failure to identify true confirmed cases may have important public health consequences.

## 1. Introduction

**Figure 1:**
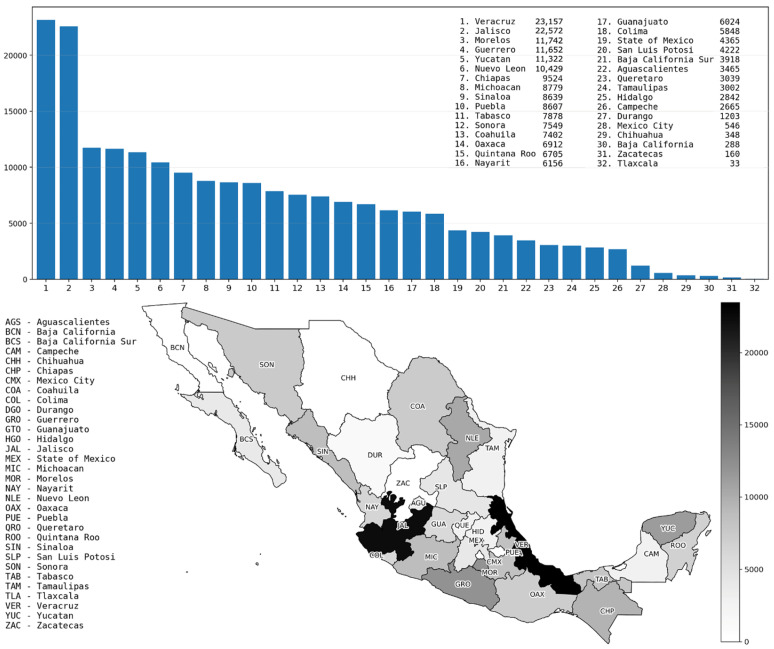
Distribution of confirmed dengue cases in Mexico by state (2021–2025).

The outbreak of arboviral diseases such as dengue represents a significant public health challenge worldwide, particularly in Latin American countries. In an epidemiological alert issued by the National Committee for Epidemiological Surveillance of Mexico [1], it was reported that 2,811,433 cases of dengue occurred in Latin America in 2022, making it the year with the third highest number of cases in this region, surpassed only by 2016 and 2019. However, by mid-2023, a total of 3,034,889 dengue cases had been reported, with a cumulative incidence rate of 305 cases per 100,000 inhabitants, exceeding the total number of cases registered during the entirety of 2022. The alert also highlights that dengue fever has a seasonal pattern: in the Southern Hemisphere, most cases occur during the first half of the year, while in the Northern Hemisphere (where Mexico is located), cases occur mostly in the second half of the year. This pattern corresponds with the warmest and rainiest months in tropical and subtropical regions of the world. Another entity that provides information on dengue fever in Latin America is the Pan American Health Organization (PAHO) [2]; their reports show that Brazil and Mexico have the highest number of reported dengue cases in this region and that four dengue serotypes (DENV1, DENV2, DENV3, and DENV4) are present in these two countries. In Mexico, the General Epidemiology Directorate [3] has published more specific information on dengue fever, including data related to the different Mexican states. Figure 1 shows the distribution of the 210,993 confirmed dengue cases among these different states throughout January 2021 to December 2025, with Veracruz and Jalisco standing out as having the highest numbers of cases (23,157 and 22,572, respectively).

An interesting characteristic of the states of Veracruz and Jalisco is their geographical separation (marked in black on the map, generated by ChatGPT 5.2). Veracruz is located on the Atlantic coast (Gulf of Mexico), and Jalisco is on the Pacific coast; however, a shared characteristic is that a significant portion of their territory has a tropical climate. Meanwhile, the states with the lowest number of confirmed cases are landlocked, except for Baja California. This is the case for Tlaxcala, Zacatecas, Chihuahua, and Mexico City, regions located in central and northern Mexico. Nonetheless, there are 10 Mexican states that also do not have a coastline but have a significant number of confirmed cases, namely Morelos, Nuevo Leon, Coahuila, Guanajuato, State of Mexico, San Luis Potosí, Aguascalientes, Queretaro, Hidalgo, and Durango.

Based on this data, the aim of our work was to analyze the behavior of dengue in Mexico over the last five years, focusing on 2025, to develop binary classification models regarding confirmed and negative dengue cases, employing machine learning (ML) algorithms and the Python programming language version 3.13.7. Furthermore, as an important part of our work, we reviewed various related studies, both from Mexico and other parts of the world, which are described below.

First, we detail the work carried out in the context of Mexico. Appice et al. [4] proposed a multi-stage machine learning strategy (AutoTiC-NN) that combines auto-encoding, window-based data slicing and cluster analysis to uncover the temporal dynamics in historical measurements of temperature and dengue variables. They used the knowledge obtained from their strategy to develop a time-series Nearest Neighbor predictor, applying it to dengue case records collected monthly over the period of 1985–2010 in several Mexican states. According to the authors, their proposed method had better predictive accuracy than other competitor methods and can be employed to forecast future dengue outbreaks. Baak-Baak et al. [5] performed a spatio-temporal analysis of dengue cases and deaths in Mexico from 2007 to 2020, using ML to cluster Mexican states according to whether there was a low risk (Cluster 1), moderate risk (Cluster 2), or high risk (Cluster 3) of dengue to select indices associated with this disease. The authors suggested designing strategies to mitigate the burden of dengue in Cluster 3, composed mainly of coastal states in the southeast, southwest, and west of Mexico, since, according to their results, this cluster contributed 71.4% of confirmed cases and 89.2% of deaths. Dong et al. [6] presented an integrated analysis of *Aedes*-borne diseases (ABDs), the local climate, and the socio-demographic profiles of 2469 municipalities in Mexico over the period of January 2012–December 2019. They compared six ML techniques, namely XGBoost, Decision Trees, Support Vector Machine, k-Nearest Neighbors, Random Forest, and neural networks, to predict ABD prevalence and found that XGBoost performed the best for this purpose. Furthermore, they determined that socio-economic attributes had a greater influence than climatic attributes on predicting the ABD prevalence and that dengue was the most prevalent disease across Mexico, with nearly 60.6% of municipalities reporting cases.

Relevant studies have also been carried out in the Latin American context. Brazil stands out as a case study of great importance, as it leads the region in terms of dengue cases. Some interesting studies using ML techniques are those of Sebastianelli et al. [7], Santos et al. [8], and da Silva et al. [9]. In the first of these studies, an ensemble model for forecasting the dengue incidence rate (DIR) in Brazil is proposed. This model integrates spatial and temporal information from multiple sources, including earth observation satellite products, climate reanalysis models, socio-economic variables, and geospatial features for the period of 2001–2019, providing one-month-ahead DIR estimates at the state level using an ML framework featuring Categorical Boosting, Support Vector Machine, and Long Short-Term Memory. According to the authors, their model showed good accuracy across the 27 Brazilian Federal Units and also excellent performance when applied to another country (Peru). In the second study, an ML model was developed to estimate potential misdiagnosed dengue hospitalizations in Brazil using data from the Brazilian public healthcare system and the National Institute of Meteorology. The ML algorithms used in the model were Random Forest, Logistic Regression, and Support Vector Machine. According to the authors, the best model was Random Forest, with an accuracy of 85% on the final reviewed test, showing that 3.4% of all hospitalizations (13,608) in the public healthcare system from 2014 to 2020 could have been dengue misdiagnosed as other diseases. In the third study, the authors compared a region of Brazil with two other regions of South America. They conducted a study on dengue forecasting using the Random Forest algorithm on data from the three regions to identify which variables most influenced the forecast in each region. They used dengue data from Natal, Brazil; Iquitos, Peru; and Barranquilla, Colombia, from 2016 to 2019, 2001 to 2012, and 2011 to 2016, respectively. According to the authors, for Natal, the best forecast was achieved using dengue cases without considering climatic and humidity data, while in the case of Iquitos and Barranquilla, the results were improved by including climatic and humidity data, respectively. The authors presented various time-series graphs that demonstrate the performance of the Random Forest algorithm in terms of the error between the real and simulated data and other metrics for the three cases.

Other relevant studies related to Latin American countries are those presented by Madewell et al. [10], Gupta et al. [11], and Exebio-Chepe et al. [12]. In the first of these studies, the authors trained and evaluated nine ML models—Decision Trees, K-Nearest Neighbors, Naïve Bayes, Support Vector Machine, artificial neural networks, AdaBoost, CatBoost, LightGBM, and XGBoost—to predict severe dengue in Puerto Rico using data from the Sentinel-Enhanced Dengue Surveillance System over the period of May 2012–August 2024. According to the authors, among the 1708 confirmed dengue cases, 24.3% were classified as severe. The Gradient Boosting algorithms achieved the highest predictive performance based on an AUC-ROC value of 97.1%. In the second study, the authors proposed a diagnostic and prognostic model for dengue disease using ML techniques, including K-Nearest Neighbors, Decision Trees, Random Forest, Support Vector Machine, and Naïve Bayes. They reported that Random Forest was the best-performing algorithm, with a mean score of 8.72 using a 10-K-fold cross-validation. In their work, a dataset of 1872 dengue cases was used, obtained from the website DengAI: Predicting Disease Spread [13], which contains information on dengue fever cases reported in San Juan, Puerto Rico, and Iquitos, Peru. In the third study, the authors performed a comparative analysis of the Support Vector Machine, Random Forest, and artificial neural network ML algorithms for dengue virus classification using a dataset of 21,157 cases from a public hospital in Lambayeque, Peru, over January–July 2023; 70% was used for training and 30% for testing. They report that the artificial neural network demonstrated the best performance, with 86.47% accuracy and 92.91% Recall in classifying dengue-related cases.

Studies on dengue have also been carried out using ML in other regions of the world. Tian et al. [14] proposed an ML approach for predicting dengue fever in Singapore that incorporates meteorological data from 2012 to 2022. They assessed various ML algorithms using the Mean Absolute Error (MAE), Root Mean Square Error (RMSE), and R-square (R^2^) performance metrics. The authors report that the XGBoost model showed the best performance, with MAE = 89.12, RMSE = 156.07, and R^2^ = 0.83. Huang et al. [15] also developed ML models for assessing the risk of dengue using a dataset of 1581 patients at National Cheng Kung University Hospital in Taiwan. The authors report that the artificial neural network model showed the highest average discrimination area under the receiver operating characteristic curve (ROC-AUC), with a value of 0.8324, and a balance accuracy of 0.7523. Salim et al. [16] evaluated ML models for predicting dengue outbreaks in the state of Selangor, Malaysia, using a dataset of notified cases with dengue clinical symptoms over 2013–2017. The authors report that the Support Vector Machine model showed the best prediction performance, with accuracy = 70%, sensitivity = 14%, specificity = 95%, and Precision = 56%. Yavari Nejad and Varathan [17] presented another study conducted in Malaysia, which evaluated five ML models for predicting dengue outbreaks using data on confirmed dengue cases between January 2010 and December 2013 published by the Ministry of Health Malaysia. The models evaluated were Bayes Network, Support Vector Machine, RBF Tree, Decision Table, and Naïve Bayes. The authors identified the most important climatic factors that contribute to dengue outbreaks and their experimental results showed that the Bayes Network model had an accuracy of 92.35%.

## 2. Materials and Methods

This study used dengue data published by the Mexican government on dengue [3] from January 2021 to December 2025 (focusing on 2025), which were preprocessed before analysis using ML techniques. Table 1 shows the 28 variables in the Mexican government’s dengue dataset, including administrative, chronological, clinical, comorbidity, geographic, and socio-demographic variables.

**Figure 2:**
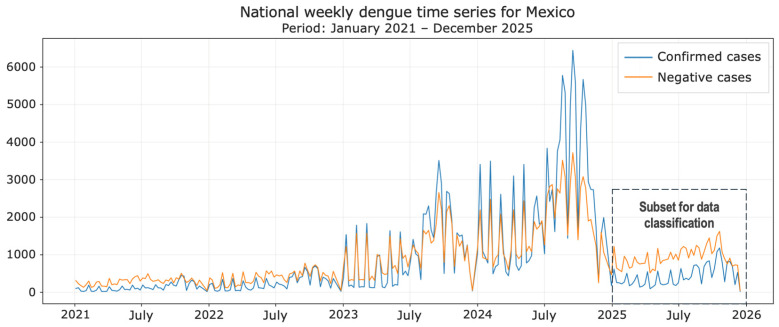
Confirmed and negative dengue cases in Mexico (2021–2025).

A preliminary analysis of the dengue dataset shows that dengue remains a major public health issue in Mexico, especially from 2023 to the present, as can be seen in the time series shown in Figure 2. Particularly, in 2024, there was an alarming increase in confirmed cases, and, as in 2023 and 2025, the greatest increase was observed during the hottest periods (summer).

**Figure 3:**
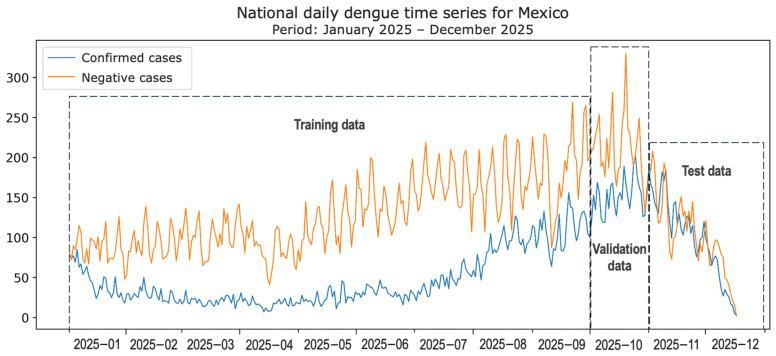
Dengue dataset windows for training, validating and testing classification models.

Considering the significant variability in the number of cases throughout 2021–2025, as observed in Figure 2, we focused our interest mainly on the January–December 2025 period to perform a binary classification of suspected dengue cases. Figure 3 shows the 2025 data windows considered.

This dataset was used because machine learning models developed using more recent data exhibit less model drift and, therefore, yield more reliable predictions [18,19]. In other words, models trained with older data have a higher risk of poor performance that does not reflect reality. Table 2 presents the number of cases in 2025 used for training, validation, and testing of the classification models, with approximately 70% of the data for training and the remaining 30% for validation and testing.

**Figure 4:**
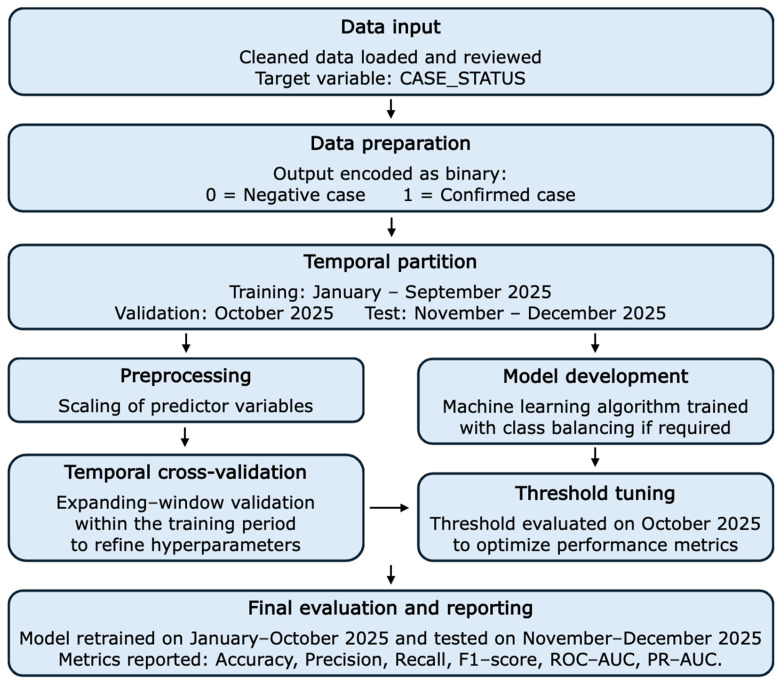
Methodology for model development and evaluation.

Regarding the methodology, Figure 4 shows the workflow followed in this study. First, the output variable was CASE_STATUS, coded as 0 for a “Negative case” and 1 for a “Confirmed case”. The variable DATE_OF_SYMPTOMS was used exclusively to establish temporal segmentation and was not included as a predictor.

The 2025 dataset was divided into 47,406 records for training (January–September), 11,202 for validation (October), and 9614 for testing (November–December). Our study included nine ML models: Random Forest, Bayesian Network, XGBoost, CatBoost, Naïve Bayes, Logistic Regression, MultiLayer Perceptron (MLP), Support Vector Machine (SVM), and LightGBM. In all cases, threshold-dependent metrics (accuracy, Precision, Recall, and F1-score) and probabilistic ranking-based metrics (ROC-AUC and PR-AUC) were analyzed [20,21].

During the preprocessing stage, Min–Max scaling was used as a recurring element to homogenize (normalize) the values of several variables, especially the AGE variable. Additionally, for some algorithms, one-hot coding was applied to categorical variables (Logistic Regression and Support Vector Machine), and in the case of the Bayesian Network model, the AGE variable was discretized by quantiles to fit the completely discrete structure.

The treatment of imbalance (in the training dataset) also varied among the different models. For example, Class Weighting was used in the development of the Random Forest model, considering that the positive class (“Confirmed case”) was the minority [22,23], while UnderSampling was used for XGBoost. In addition, RandomOverSampler and configurations with and without balancing were used as part of model training.

The importance of variables was estimated using different strategies depending on the model: native importance in the Random Forest and Gradient Boosting models, aggregated coefficients in Support Vector Machine, permutation importance in Logistic Regression and MultiLayer Perceptron, and mutual information in the Bayesian Network and Naïve Bayes models [24,25]. At this point, it is important to note that some variables were not considered a priori in our study due to their irrelevance, either because they are basically administrative variables (UPDATE_DATE and RECORD_ID), or because they are misleading variables that could introduce information leakage by not being available at the prediction time (PATIENT_TYPE, DEATH, DIAGNOSIS, SAMPLE_COLLECTION, and PCR_RESULT).

Figure 4 provides an integrated summary of the procedure logic used, showing that the model selection was not based on a random partition, but rather on a temporal scheme consistent with the data’s nature. It also shows that temporal cross-validation and adjustment of the decision threshold were complementary: the goal of the former was to refine model hyperparameters, and that of the latter was to optimize model operational performance in terms of the accuracy, Precision, Recall, and F1-score metrics.

## 3. Results

Table 3 presents the metrics resulting from the application of nine ML algorithms to the dataset of 68,222 suspected dengue cases in 2025. The metrics were obtained based on a threshold aimed at maximizing the F1-score, seeking a balance between the maximum detection of true positive cases (confirmed cases) and the minimum prediction of false positives; that is, a balance between the Precision and Recall metrics, considering that the training dataset is significantly unbalanced.

Table 4 presents a summary analysis of the most influential variables during the development of the different classification models. These results were obtained using Native Feature Importance for Random Forest, XGBoost, CatBoost, LightGBM, and Logistic Regression. For Bayesian Network and Naïve Bayes, the non-parametric mutual information method was used, while for MLP and SVM, permutation importance and the absolute value of the model coefficients were used, respectively.

The metrics presented in Table 3 were obtained by analyzing different thresholds, with the aim of achieving the best overall balance in model performance along with greater recovery of true positive cases, while avoiding a significant decrease in overall performance. As an example, Table 5 presents a comparison of three thresholds for the Random Forest model, in both the validation and testing phases. The first threshold is the default threshold (0.5), the second is focused on the overall performance balance (0.397), and the third is focused on greater recovery of true positive cases (0.327). The same procedure was followed for the other models.

**Figure 5:**
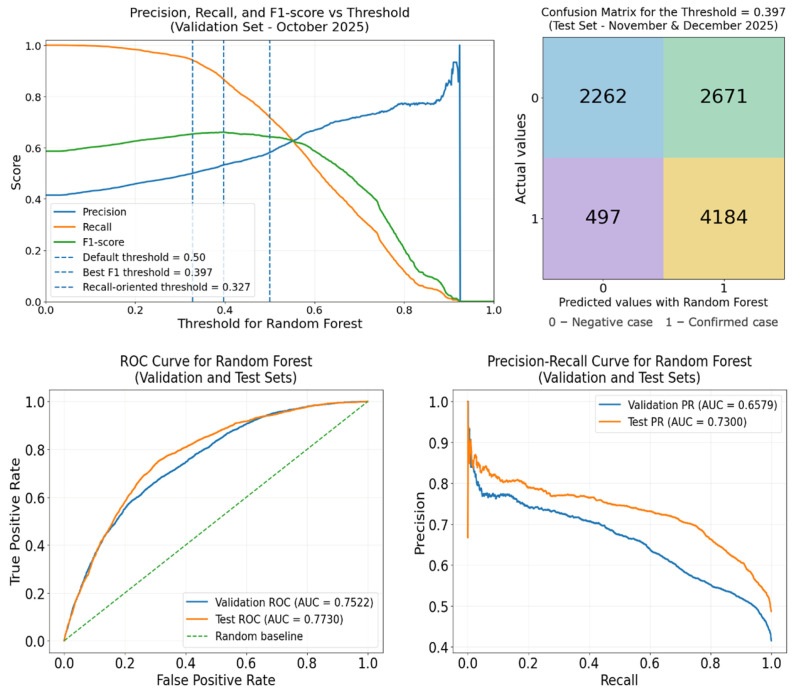
Threshold analysis, confusion matrix, ROC curve, and PR curve for the RF model.

As a complement to Table 5, Figure 5 shows a set of graphs and the confusion matrix corresponding to the optimal threshold (0.397) in the testing phase for the Random Forest model. One graph shows the curves for the Precision, Recall, and F1-score metrics for a comparative analysis of threshold behavior, particularly for the three thresholds mentioned above. The other two graphs correspond to the ROC-AUC and PR-AUC, which show that, in general, these metrics improve during the testing phase.

To provide greater certainty for the metric values obtained with the optimal threshold for the Random Forest classifier, Table 6 presents the 95% confidence interval for each value.

These confidence intervals were estimated using the non-parametric bootstrap technique, applied separately to the validation and test sets. In each bootstrap iteration, a sample (of the same size and with replacement) was generated, and the metrics were calculated using the 0.397 threshold. Thus, the metrics based on binary classification (accuracy, Precision, Recall, and F1-score) were calculated from the predicted classes (0/1), obtained after applying this threshold, while the metrics based on discrimination (ROC-AUC and PR-AUC) were calculated from the probabilities predicted by the RF model. Finally, the 95% confidence intervals were obtained from the 2.5 and 97.5 percentiles of the empirical distribution generated by the resamples.

## 4. Discussion

Table 3 shows that the models with the best overall balance were Random Forest, CatBoost, and Logistic Regression. The former stands out for achieving the best F1-score and the best PR-AUC, in addition to leading to a significant operational improvement in Recall with minimal F1-score cost. On the other hand, the Bayesian Network, XGBoost, CatBoost, Logistic Regression, Support Vector Machine, and LightGBM models achieved solid results, although they did not consistently outperform Random Forest. The Bayesian Network achieved the best ROC-AUC, although this metric has less informational weight compared to PR-AUC for unbalanced datasets [20]. The Naïve Bayes case can be considered the least significant, even though it achieved the best Recall, but at a very high cost in other metrics.

Regarding the threshold adjustment, its contribution was not homogenous. That is, in some models, especially in Random Forest, it was clearly useful since it shifted the classifier toward a much better detection of true positive cases (Recall), but in other models, such as XGBoost and SVM, the observed improvement in validation did not translate into a clear gain in testing.

Another relevant finding of our study was the consistency of the results in variable importance, as the locations of residence and the notifying medical unit proved more influential than most comorbidities across the different models. This suggests that the CASE_STATUS classification depends not only on individual attributes but also on the territorial and operational surveillance system contexts. Nonetheless, this conclusion should be interpreted in predictive rather than causal terms, since the variable importance in linear, tree, or permutation models does not necessarily imply an explanation of the causes of a disease [25,26].

Specifically, given that the main objective of this work was to maximize the overall balance of model performance at the same time as achieving a high detection rate for real confirmed cases, the Random Forest model emerged clearly as the most robust alternative, although there is always the possibility of improvement with more up-to-date data in the near future, just like with the other models. The obtained results show that the Random Forest model performed well in classifying CASE_STATUS within a realistic timeframe. Validation and testing in subsequent months allowed for a more robust estimate of model generalization, avoiding artificially optimistic assessments. This approach is consistent with best practices in supervised modeling and predictive evaluation [26,27]. Furthermore, temporal cross-validation was useful for refining the forest configuration and led to a slightly regularized version of the model (min_samples_leaf changed from 2 to 3), which improved performance stability during validation and testing. In addition, adjusting the threshold allowed the classifier’s behavior to be adapted to the analytical objective: maximizing the F1-score or prioritizing Recall.

Taken together, the graphical and quantitative evidence allows us to conclude that the Random Forest model demonstrates solid performance for binary classification of the CASE_STATUS feature, particularly when prioritizing the detection of confirmed cases. Adjusting the decision threshold proved crucial, as it revealed that a value of 0.397 offers the best compromise between Precision and Recall; lower thresholds can be used when prioritizing sensitivity. Nonetheless, the model still exhibits limited capacity to reduce false positives, so its use should be interpreted within the operational context of the problem. That is, if the priority is minimizing undetected confirmed cases, the observed performance is favorable; however, if the cost of reviewing false positives is high, exploring additional calibration strategies, threshold adjustments, or model improvements in the future may be required.

The confidence intervals obtained for the performance metrics indicate that the Randon Forest model exhibits a consistent and statistically stable performance in both validation and testing. In particular, the observed Recall values remained high in both sets, with relatively narrow intervals, suggesting that the model maintains a robust capacity to identify confirmed cases using the optimal threshold (0.397). The F1-score, as a harmonic measure between Precision and Recall, also showed limited variability in both evaluation sets, suggesting that the balance achieved between these two metrics does not depend critically on small sample fluctuations. Meanwhile, the ROC-AUC and PR-AUC intervals also remained at acceptable values (generally above 0.7), confirming that the model maintains reasonable performance by focusing on the positive class. Furthermore, a methodically important finding is that the intervals estimated in the testing set were comparable to those observed in validation and did not show an abrupt deterioration in performance. This result suggests that the model maintains its temporal generalizability and demonstrates that the time-splitting, temporal cross-validation, threshold-adjustment strategy was appropriate for reducing the risk of overfitting. In conclusion, the 95% confidence intervals support the model’s robust performance over the analyzed period.

### Limitations

In future work, this study could be improved through a real-time data integration strategy and through developing pre-trained models that can quickly adapt to the behavior of infections. In this regard, Yu et al. [28] proposed a digital contact tracing method to address epidemic tracking using a graph neural network, which combines forward and backward tracing to identify potential sources of infection or superspreaders in a contact network. This method is used for person-to-person transmitted diseases (evaluated using COVID-19 data) and could be adapted for mosquito-borne diseases, such as dengue, by replacing the human contact graph with a heterogeneous spatio-temporal graph featuring people, households, workplaces, schools, neighborhoods, mosquito breeding sites, and vector surveillance units as nodes.

## 5. Conclusions

This study demonstrates that temporally structured machine learning models can be effectively used to classify suspected dengue cases in Mexico from national surveillance data. By preserving the chronological order of the data during training, validation, and testing, a realistic assessment of predictive performance under conditions that more closely resemble real-world epidemiological use was achieved using the proposed approach.

Among the nine evaluated algorithms, Random Forest emerged as the most robust model, mainly because it achieved the best balance between sensitivity and global predictive performance, as reflected by its F1-score and PR-AUC values. In practical terms, this is especially relevant in dengue surveillance, where failing to identify true confirmed cases may have important public health implications. Adjusting the decision threshold further improved the operational value of the model by allowing its performance to be tuned according to the analytical priority.

An additional relevant finding was the consistency of the feature-importance results across models. Variables related to territorial context and to the notifying medical unit contributed more strongly than most comorbidities, suggesting that dengue case classification is influenced not only by patient-level characteristics but also by geographic and institutional patterns embedded in the surveillance system.

Overall, the findings support the use of recent, temporally validated, and threshold-optimized machine learning models as complementary tools for dengue surveillance in Mexico. Nevertheless, the persistence of false positives indicates that further work is needed to improve model calibration, optimize threshold selection under different operational scenarios, and incorporate newly available data to maintain predictive reliability over time.

## Figures and Tables

**Table 1:** Variables defined by the Mexican government in the published dengue dataset.

No.	Variable Name	Description
1	UPDATE_DATE	This variable identifies the latest update date (the database is updated weekly).
2	RECORD_ID	Case identifier number.
3	SEX	This variable identifies the patient’s sex.
4	AGE	This variable identifies the patient’s age in years.
5	RESIDENCE_STATE	This variable identifies the patient’s residential state.
6	RESIDENCE_MUNICIPALITY	This variable identifies the patient’s residential municipality.
7	INDIGENOUS_LANGUAGE_SPEAKING	This variable identifies if the patient speaks an indigenous language.
8	INDIGENOUS	This variable identifies if the patient self-identifies as an indigenous person.
9	NOTIFYING_MEDICAL_UNIT_ENTITY	This variable identifies the state where the notifying medical unit is located.
10	NOTIFYING_MEDICAL_UNIT_MUNICIPALITY	This variable identifies the municipality where the notifying medical unit is located.
11	NOTIFYING_MEDICAL_UNIT_INSTITUTION	This variable identifies the notifying medical unit institution.
12	DATE_OF_SYMPTOMS	This variable identifies the onset date of signs and symptoms of the current condition.
13	PATIENT_TYPE	This variable identifies the type of care the patient received in the medical unit. It is “outpatient” if they returned home, or “hospitalized” if they were admitted to the hospital.
14	BLEEDING_DISORDERS	Presence of bleeding disorders.
15	DIABETES	Presence of diabetes.
16	HYPERTENSION	Presence of hypertension.
17	PEPTIC_ULCER_DISEASE	Presence of peptic ulcer disease.
18	KIDNEY_DISEASE	Presence of kidney disease.
19	IMMUNOSUPPRESSION	Presence of immunosuppression.
20	LIVER_CIRRHOSIS	Presence of liver cirrhosis.
21	PREGNANCY	This variable identifies if the patient (female) is pregnant.
22	DEATH	This variable indicates if the patient died.
23	DIAGNOSIS	This variable identifies the assessment outcome for the patient.
24	SAMPLE_COLLECTION	This variable identifies if a sample was taken from the patient.
25	PCR_RESULT	This variable identifies the result of the PCR test performed by the laboratory.
26	CASE_STATUS	This variable identifies the status of the case.
27	ASSIGNED_STATE	This variable identifies the state to which the case was assigned.
28	ASSIGNED_MUNICIPALITY	This variable identifies the municipality to which the case was assigned.

**Table 2:** Dengue dataset periods used for model training, validation and testing.

Dataset	Period	Dengue Cases	Percentage
Training	January–September 2025	35,003 Negative cases 12,403 Confirmed cases	69.5%
Validation	October 2025	6553 Negative cases 4649 Confirmed cases	16.4%
Test	November–December 2025	4681 Negative cases 4933 Confirmed cases	14.1%
Total	January–December 2025	68,222 total cases	100%

**Table 3:** Validation and testing metrics of the developed classification models.

ML Model	~16% + 14%	Accuracy	Precision	Recall	F1-Score	ROC-AUC	PR-AUC	Summary Analysis
Random Forest Threshold = 0.397	Validation Test	0.6303 0.6705	0.5336 0.6104	0.8679 0.8938	0.6609 0.7254	0.7522 0.7730	0.6579 0.7300	Best F1-score and PR-AUC. Second-best Recall and ROC-AUC.
Bayesian Network Threshold = 0.1853	Validation Test	0.7179 0.7023	0.6120 0.6056	0.7985 0.7897	0.6929 0.6855	0.7900 0.7756	0.6796 0.6724	Best ROC-AUC and accuracy. ROC-AUC similar to Random Forest.
XGBoost Threshold = 0.507	Validation Test	0.6858 0.6834	0.5897 0.6428	0.7982 0.7870	0.6783 0.7076	0.7660 0.7496	0.6650 0.7099	Good performance across all metrics, but none of them are the best.
CatBoost Threshold = 0.47	Validation Test	0.6453 0.6630	0.5485 0.6104	0.8228 0.8511	0.6582 0.7109	0.7428 0.7436	0.6450 0.7023	Good performance across all metrics, but none of them are the best.
Logistic Regression Threshold = 0.49	Validation Test	0.6730 0.6830	0.5742 0.6326	0.8202 0.8321	0.6755 0.7188	0.7511 0.7445	0.6368 0.6936	Good performance across all metrics, but none of them are the best.
Naïve bayes Threshold = 0.001	Validation Test	0.4052 0.4199	0.4003 0.4135	0.9876 0.9848	0.5697 0.5825	0.5341 0.5615	0.4534 0.4738	Best Recall, but the other metrics are the worst. Poor performance.
MLP Threshold = 0.5	Validation Test	0.6261 0.6245	0.5230 0.5309	0.7082 0.7407	0.6017 0.6185	0.6964 0.7001	0.6023 0.6106	Some metrics are relatively good, and others are a little low.
SVM Threshold = 0.0	Validation Test	0.6937 0.6909	0.5847 0.5929	0.7996 0.7900	0.6755 0.6774	0.7726 0.7682	0.6521 0.6597	Good performance across all metrics, except for Precision.
LightGBM Threshold = 0.3174	Validation Test	0.6980 0.6906	0.6030 0.6543	0.7969 0.7727	0.6866 0.7086	0.7711 0.7452	0.6696 0.7000	Best Precision and good performance in other metrics, but still below other models.

**Table 4:** Important patterns of recurring variables in the nine models.

Recurring Pattern	Models in Which It Was Identified	Conclusive Analysis
State and municipality of residence	RF, BN, XGBoost, CatBoost, LR, NB, MLP, SVM, LightGBM.	The classification is associated with the territorial context.
State and municipality of the notifying medical unit	RF, BN, XGBoost, CatBoost, LR, NB, MLP, SVM, LightGBM.	The classification is associated with the operational and institutional context.
Age	RF, BN, XGBoost, CatBoost, LR, NB, MLP, SVM, LightGBM.	It provides additional, but usually secondary, information.
Comorbidities	Mainly XGBoost, MLP, and SVM; minor contribution in other models.	Its contribution was weak in most models.

**Table 5:** Metrics of default, best F1 and Recall-oriented thresholds for the RF model.

Random Forest						
VALIDATION (October 2025)						
Threshold	Accuracy	Precision	Recall	F1-score	ROC-AUC	PR-AUC
0.500	0.6683	0.5812	0.7182	0.6425	0.7522	0.6579
0.397	0.6303	0.5336	0.8679	0.6609	0.7522	0.6579
0.327	0.5851	0.5001	0.9426	0.6534	0.7522	0.6579
TEST (November–December 2025)						
Threshold	Accuracy	Precision	Recall	F1-score	ROC-AUC	PR-AUC
0.500	0.7069	0.6668	0.7956	0.7255	0.7730	0.7300
0.397	0.6705	0.6104	0.8938	0.7254	0.7730	0.7300
0.327	0.6252	0.5699	0.9387	0.7092	0.7730	0.7300

**Table 6:** Confidence intervals for the RF metric values (threshold = 0.397).

Random Forest				
Metric	Validation (October 2025)		Test (November–December 2025)	
	Value	CI 95%	Value	CI 95%
Accuracy	0.6303	0.6214–0.6392	0.6705	0.6610–0.6798
Precision	0.5336	0.5223–0.5448	0.6104	0.5988–0.6218
Recall	0.8679	0.8579–0.8774	0.8938	0.8847–0.9023
F1-score	0.6609	0.6511–0.6705	0.7254	0.7161–0.7346
ROC-AUC	0.7522	0.7428–0.7616	0.7730	0.7636–0.7824
PR-AUC	0.6579	0.6410–0.6643	0.7300	0.7222–0.7472

## Data Availability

The data used for this research are available for download on the website of the General Epidemiology Directorate of Mexico, at https://www.gob.mx/salud/documentos/datos-abiertos-bases-historicas-de-enfermedades-transmitidas-por-vector (accessed on 6 January 2026).

## Abbreviations

The following abbreviations are used in this manuscript:

ABD	*Aedes*-Borne Disease
AdaBoost	Adaptive Boosting
ANN	Artificial Neural Network
auto.arima	Automatic AutoRegressive Integrated Moving Average
BN	Bayes Network
CatBoost	Categorical Boosting
CI	Confidence Interval
DENV	Dengue Virus
DT	Decision Tree
GB	Gradient Boosting
GLM	General Linear Model
kNN	k-Nearest Neighbor
LightGBM	Light Gradient Boosting Machine
LR	Logistic Regression
LSTM	Long Short-Term Memory
M5	M5 Model Tree
MAE	Mean Absolute Error
ML	Machine Learning
MLP	MultiLayer Perceptron
NB	Naïve Bayes
nRMSE	Normalized Root Mean Square Error
PR-AUC	Area Under the Precision-Recall Curve
R	Correlation Coefficient
R^2^	Determination Coefficient
RBF	Radial Basis Function
RF	Random Forest
RMSE	Root Mean Square Error
ROC-AUC	Area Under the Receiver Operating Characteristic Curve
SVM	Support Vector Machine
SVR	Support Vector Regression
TS	Time Series
VAR	Vector AutoRegression
XGBoost	xTreme Gradient Boosting
